# An Electrochemical Sensor Based on Three-Dimensional Porous Reduced Graphene and Ion Imprinted Polymer for Trace Cadmium Determination in Water

**DOI:** 10.3390/s23239561

**Published:** 2023-12-01

**Authors:** Linzhe Wang, Jingfang Hu, Wensong Wei, Shuyu Xiao, Jiyang Wang, Yu Song, Yansheng Li, Guowei Gao, Lei Qin

**Affiliations:** 1 Beijing Key Laboratory of Sensor, Beijing Information Science & Technology University, Beijing 100101, China; wlz_98@163.com (L.W.); shanshui4956@126.com (Y.L.);; 2 Institute of Food Science and Technology, Chinese Academy of Agricultural Sciences/Key Laboratory of Agricultural Product Processing, Ministry of Agriculture, Beijing 100193, China; 3 Key Laboratory of Modern Measurement and Control Technology, Ministry of Education, Beijing Information Science & Technology University, Beijing 100192, China; 4 State Key Laboratories of Transducer Technology, Shanghai Institute of Microsystems and Information Technology, Chinese Academy of Sciences, Shanghai 200050, China; 5 Zibo Institute for Digital Agriculture and Rural Research, Zibo 255051, China

**Keywords:** Cd(II) determination, ion-imprinted polymer, three-dimensional porous graphene, electrochemical sensor

## Abstract

Three-dimensional (3D) porous graphene-based materials have displayed attractive electrochemical catalysis and sensing performances, benefiting from their high porosity, large surface area, and excellent electrical conductivity. In this work, a novel electrochemical sensor based on 3D porous reduced graphene (3DPrGO) and ion-imprinted polymer (IIP) was developed for trace cadmium ion (Cd(II)) detection in water. The 3DPrGO was synthesized in situ at a glassy carbon electrode (GCE) surface using a polystyrene (PS) colloidal crystal template and the electrodeposition method. Then, IIP film was further modified on the 3DPrGO by electropolymerization to make it suitable for detecting Cd(II). Attributable to the abundant nanopores and good electron transport of the 3DPrGO, as well as the specific recognition for Cd(II) of IIP, a sensitive determination of trace Cd(II) at PoPD-IIP/3DPrGO/GCE was achieved. The proposed sensor exhibited comprehensive linear Cd(II) responses ranging from 1 to 100 μg/L (R2 = 99.7%). The limit of detection (LOD) was 0.11 μg/L, about 30 times lower than the drinking water standard set by the World Health Organization (WHO). Moreover, PoPD-IIP/3DPrGO/GCE was applied for the detection of Cd(II) in actual water samples. The satisfying recoveries (97–99.6%) and relative standard deviations (RSD, 3.5–5.7%) make the proposed sensor a promising candidate for rapid and on-site water monitoring.

## 1. Introduction

Cadmium pollution in water environments primarily originates from wastewater from mineral mining, smelting, and chemical industries [1]. The intake of a low Cd(II) concentration can even result in fatal adverse effects on living organisms. Short-term or long-term Cd(II) exposure of the human body can lead to severe diseases and cancers [2]. According to the World Health Organization, surface water’s maximum Cd(II) level is 5 μg/L, and that of drinking water is 3 μg/L [3]. Up to now, various analytical methods have been developed for Cd(II) detection, such as atomic absorption spectrometry (AAS) [4], inductively coupled plasma mass spectrometry (ICP-MS) [5], and ion chromatography [6]. Some require intricate sample preparation, costly equipment, and skilled professionals, making them both time-consuming and expensive and rendering them impractical for on-site detection. Compared with other methods, electrochemical sensors offer rapid detection, low cost, high sensitivity, and easy miniaturization. Therefore, electrochemical sensors are considered up-and-coming tools for sensitive, fast, and low-cost monitoring of heavy metals in water [7].

Three-dimensional porous graphene (3DPG) has recently attracted considerable interest as an ideal electrode material for electronic and electrochemical devices. This is attributed to its graphene composition and the precisely controlled pore structures [8]. Two-dimensional graphene always suffers from re-aggregation problems due to the strong Van der Waals interaction during charge/discharge, whereas 3DPG facilitates the rapid transport of electrons and ions and prevents agglomeration effectively. It has been widely reported that 3DPG nanostructures with large surface area and excellent electron transport can be fabricated using 2D graphene nanosheets through template-directed chemical vapor deposition (CVD) [9,10,11] or template assembly methods [12,13,14]. Template-directed CVD is the most highly efficient technology to produce 3DPG, with higher electrical conductivity and fewer defects using nickel foam as template substrate. However, it requires expensive equipment and a complicated technological process. Chen et al. [15] developed a monodisperse polymethyl methacrylate (PMMA) template assembly method to prepare macroporous “bubble” 3DPG film. However, the preparation process involved strict experimental conditions such as a vacuum and a high temperature of 800 °C. The layer-by-layer method was also employed to synthesize 3DPG material using SiO_2_ particles as sacrificial templates, where a temperature of 800 °C was also required for GO reduction and highly toxic hydrofluoric acid was necessary for template removal [14]. Therefore, the development of simple and green methods for 3DPG nanostructure fabrication is still a challenging task. On the other hand, the functionalization of 3DPG using other inorganic nanomaterials, organic polymers, or biological materials provides vast applications in supercapacitors and sensors [16,17,18].

In the field of sensors, biosensors have been developed using enzymes, DNA, antibodies, aptamers, and cells to improve the specific recognition ability of Cd(II) detection. However, these biological receptors can be easily deactivated in the presence of potential inhibitors, poisoned by sample contaminations, or limited to specific working conditions. These limitations result in poor stability when employing biosensors for on-field applications [19]. To overcome these challenges, artificial receptors of ion-imprinted polymers (IIPs)/molecularly imprinted polymers (MIPs) represent high specificity and stability for target recognition. IIPs/MIPs are synthesized through the polymerization of monomers and cross-linkers in the presence of a template, namely, the target analyst. The resulting interior cavities in the polymer skeleton are highly complementary to the template in terms of size, shape, and the multiple interactions between functional groups. These make IIPs/MIPs highly specific in recognizing the target analyst in water samples [20]. In addition, IIPs/MIPs are generally stable at various temperatures, pH, and pressure. They can also be applied in organic solvents and are much more robust than their biological materials.

Herein, we present a novel electrochemical sensor based on three-dimensional porous graphene oxide (3DPrGO) and IIP for trace Cd(II) determination in water. A 3DPrGO material was synthesized in situ on a glassy carbon electrode (GCE) surface using a PS colloidal crystal template-assisted electrochemical reduction method without any toxic regents for GO reduction or template dissolution. IIP was then modified on the 3DPrGO surface by electropolymerization with o-phenylenediamine (oPD) as a functional monomer (IIP/3DPrGO/GCE). The 3DPrGO possessing numerous active edges and active sites, combined with the high specific recognition properties of the IIP, resulted in a sensor with high sensitivity for Cd(II) detection. The detection limit reached as low as 0.11 μg/L. The results prove that the proposed IIP/3DPrGO/GCE is a promising sensor for Cd(II) sensing applications in water monitoring.

## 2. Experiment

### 2.1. Materials and Reagents

A polystyrene (PS) microsphere (1 μm in diameter) was purchased from Aladdin Company (Shanghai, China). Potassium ferricyanide (K_3_(Fe(CN)_6_)), acetic acid (CH_3_COOH), sodium acetate (CH_3_COONa), sulfuric acid, anhydrous ethanol, acetone (C_3_H_6_O), and cadmium sulfate (CdSO_4_) were purchased from Sinopharm Chemical Reagent Co., Ltd. (Shanghai, China). Potassium chloride (KCl), sodium hydroxide (NaOH), concentrated hydrochloric acid (HCl, 37%), mercury nitrate (Hg(NO_3_)_2_), magnesium sulfate heptahydrate (MgSO_4_·7H_2_O), manganese sulfate (MnSO_4_), and copper sulfate pentahydrate (CuSO_4_·5H_2_O) were purchased from Xilong Chemical Industry Co., Ltd. (Guangxi, China). Single-layer graphene oxide (GO) (50–200 nm sheet diameter) was purchased from XFNANO Co., Ltd. (Nanjing, China), and o-phenylenediamine (oPD) was purchased from Meiyimei Technology Co., Ltd. (Beijing, China). All reagents were of analytical grade and used as received without further purification. De-ionized water (conductivity < 0.1 µS/cm) was used throughout the experiment.

### 2.2. Apparatus

All electrochemical measurements were conducted using a CHI660E Electrochemical Workstation (Chenhua Instruments Co, Shanghai, China) with a standard three-electrode system at room temperature. The glassy carbon electrode (GCE) (10 mm × 10 mm × 2 mm), Pt wire, and solid-state silver/silver chloride (Ag/AgCl) were used as the working electrodes, counter electrodes, and reference electrodes, respectively. All electrodes were obtained from Aida Hengsheng Technology Development Co., Ltd. (Tianjin, China).

Morphological surface analysis was conducted in a scanning electron microscope (SEM) from ZEISS, GeminiSEM 560 (ZEISS, Oberkochen, Germany), operating in a vacuum mode.

### 2.3. Fabrication of 3D Porous Reduced Graphene-Modified Glassy Carbon Electrode (3DPrGO/GCE)

Before the modification of 3D porous reduced graphene (3DPrGO), glassy carbon electrodes (GCE) were polished using standard cleaning procedures [21]. This included polishing with a polishing cloth with 1.0 and 0.3 µm alumina powder, followed by sonication in acetone, ethanol, and deionized water successively. After drying at room temperature, the electrode surface was activated by cyclic voltammetry scans of between +1.6 V and −0.2 V in sulfuric acid (0.5 mol/L) at a scan rate of 50 mV/s until a stable curve was obtained.

Colloidal crystal templates were synthesized according to the vertical deposition method [22]. Briefly, 1 mL of polystyrene (PS) monodisperse emulsion (1%, *v*/*v*) was sonicated for 15 min and subsequently introduced into a flat-bottomed container. A 10 mm × 10 mm × 2 mm GCE was placed vertically in the container and then dried in an oven at 55 °C for 48 h. After the water evaporated, colloidal crystals were self-assembled on both sides of the GCE. The obtained electrode was designated as PS/GCE.

The 3DPrGO was synthesized via the electrodeposition method, filling the void volume in the PS templates with a GO solution. According to the previous work by our group [23], PS/GCE was immersed in a 10 mL KCl solution (0.1 mol/L) containing 2 mg/mL GO. Electrodeposition was performed using cyclic voltammetry (CV) for 7 cycles. The potential range was −1.2–0 V, and the scan rate was 50 mV/s. The current responses increased rapidly and gradually stabilized with the increased scanning cycles, indicating that graphene was filling the void volume in the PS templates. For comparison, rGO/GCE was prepared under the same conditions but without PS templates. After deposition, the 3DPrGO/PS/GCE was dried at room temperature and then calcined at 350 °C for 3 h to remove the PS templates [24].

### 2.4. Fabrication of Ion Imprinted Polymer-Modified 3D Porous Reduced Graphene Electrode (PoPD-IIP/3DPrGO/GCE)

**Scheme 1:**
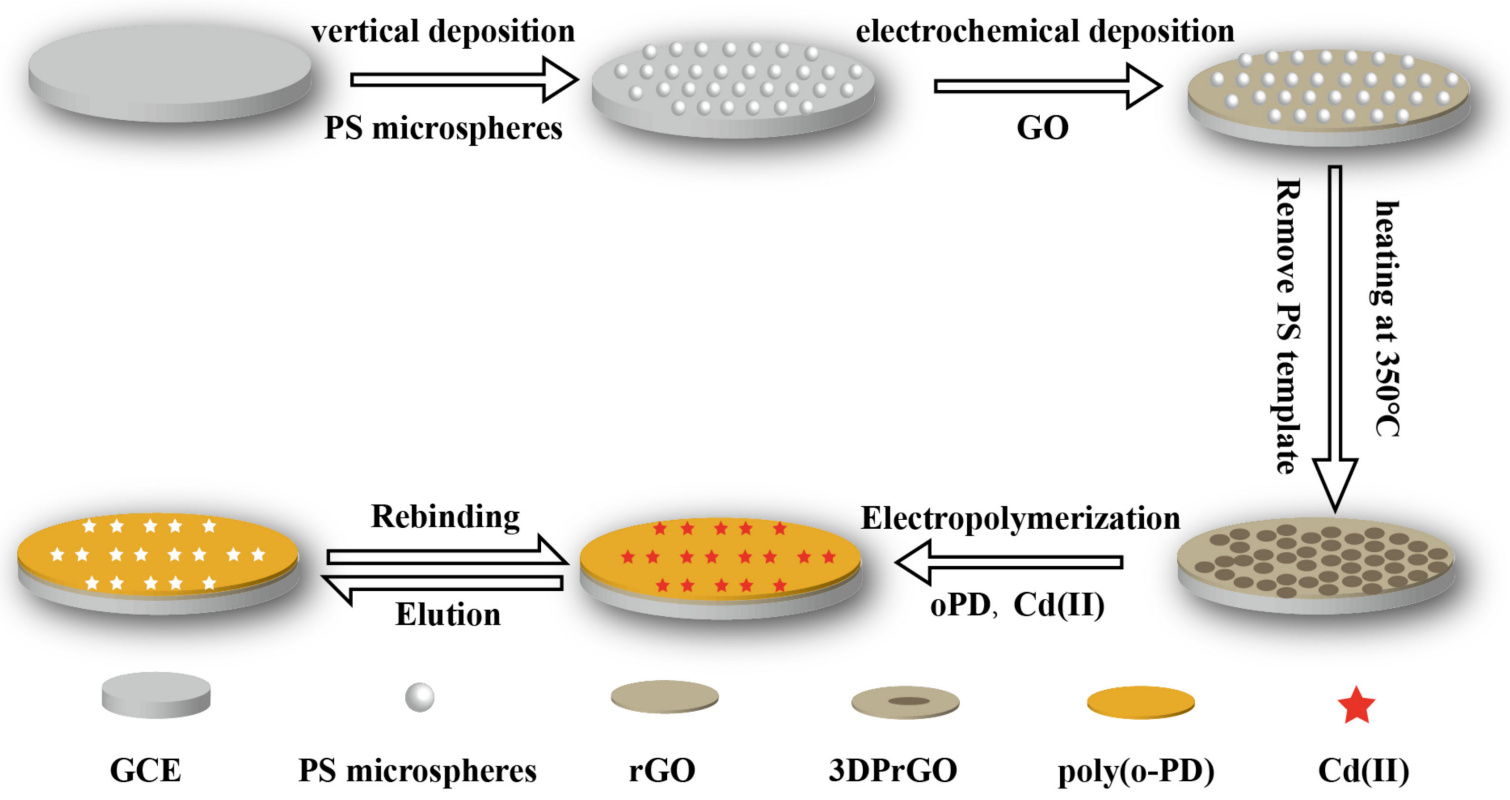
The preparation route for PoPD-IIP/3DPrGO/GCE.

The preparation process of ion-imprinted polymer-modified 3DPrGO/GCE is illustrated in Scheme 1. Electropolymerization was conducted on the surfaces of the 3DPrGO/GCE to synthesize PoPD-IIP film. This process was carried out using cyclic voltammetry (CV). Firstly, for the synthesis of Cd(II)-imprinted polymer on the 3DPrGO/GCE, a 10 mL acetate buffer solution (ABS) (0.1 mol/L, pH 5.2) containing oPD (40 mmol/L) as a functional monomer and CdSO4 (3 mmol/L) as template ions was prepared. Then, CVs (30 cycles) in the potential range of 0.0–0.8 V were performed in the above solution at a scan rate of 50 mV/s. Subsequently, the Cd(II) templates were removed via the potentiostatic method at 1.5 V in 0.1 mol/L HCL. Finally, the PoPD-IIP/3DPrGO/GCE was rinsed with de-ionized water. Furthermore, non-imprinted polymer (NIP)/3DprGO/GCE was fabricated following the same procedure but without template ions.

### 2.5. Electroanalytical Measurements

Square-wave anodic stripping voltammetry (SWASV) was carried out in a 0.1 mol/L acetate buffer solution (ABS) (pH = 4.8) containing Cd(II). The square-wave modulation in SWASV enhances the signal-to-noise ratio more than conventional voltammetry methods do, making it easier to distinguish the electrochemical signals of heavy metal ions from background noise. This allows for more sensitive measurements when testing trace levels of heavy metal ions [25]. In brief, the SWASV technique consists of three different steps. First, a −1.2 V potential was applied on the electrode surface for 400 s to preconcentrate by reducing the Cd(II) to Cd(0). Then, the anodic stripping of electrodeposited Cd(0) was carried out by applying a potential scan. The scan ranged from −1.4 to 0.2 V, with a potential step of 4 mV, a modulation amplitude of 25 mV, and a frequency of 15 Hz. Subsequently, the electrode was immersed in 0.1 mol/L HCl at a potential of +1.5 V to remove Cd(II) from ion-binding sites.

### 2.6. Real Water Sample Preparation

To assess the sensor’s suitability for real-world applications, we gathered water samples from three distinct Beijing rivers and adjusted the water samples’ pH value to 4.8. Sample analysis was performed by standard addition calibration, and the recovery rate was calculated to assess the practical accuracy of the sensor. Each group underwent three parallel experiments to ensure reliable and consistent results.

## 3. Results and Discussion

### 3.1. Characterization

**Figure 1:**
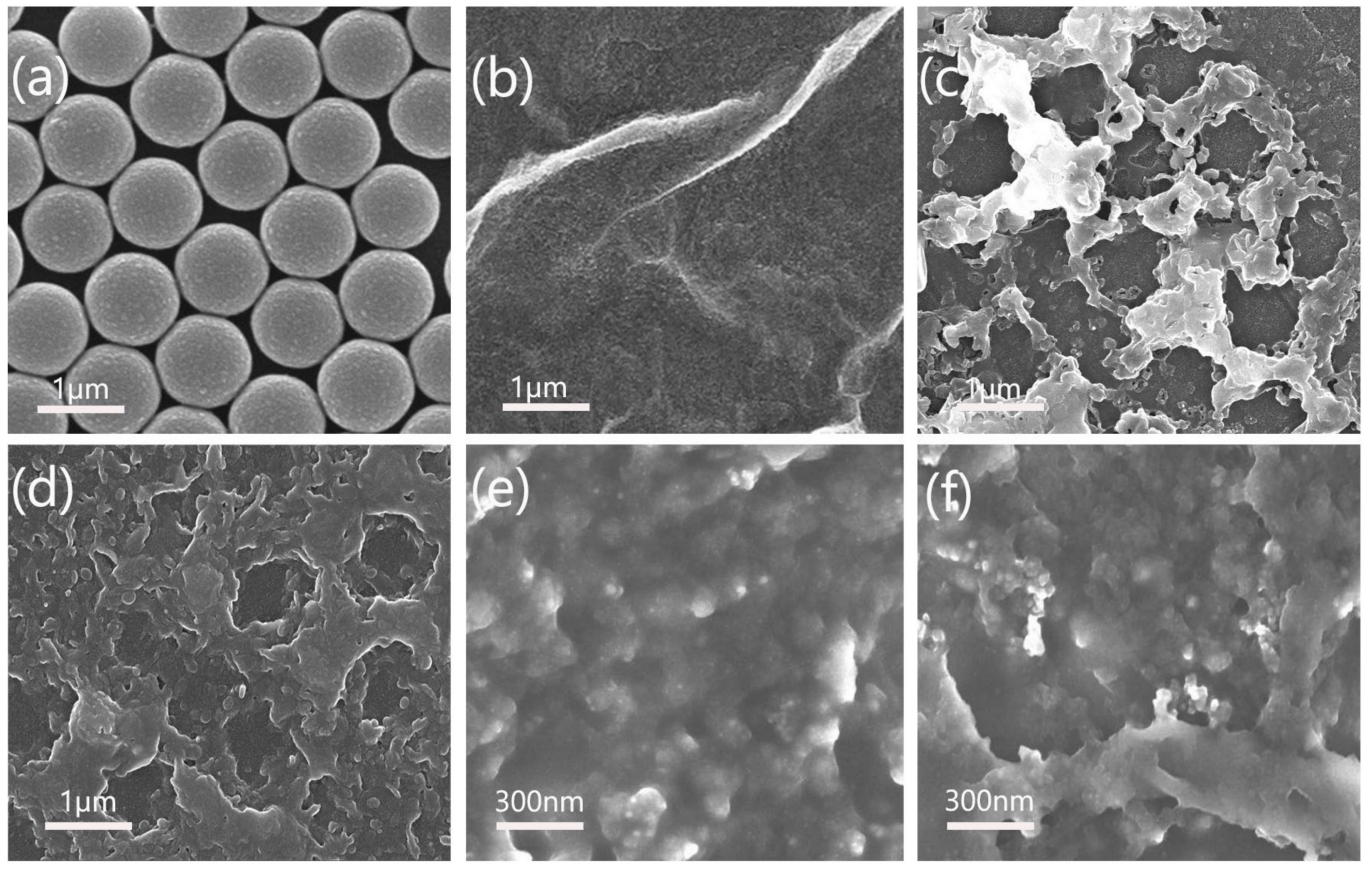
(**a**) SEM image of PS/GCE; (**b**) SEM image of rGO/GCE; (**c**) SEM image of 3DPrGO/GCE (deposition for 7 cycles); (**d**) SEM image of 3DPrGO/GCE (deposition for 9 cycles); (**e**) SEM image of PoPD-IIP/3DPrGO/GCE (before elution); (**f**) SEM image of PoPD-IIP/3DPrGO/GCE (after elution).

The morphologies of PS/GCE, rGO/GCE, 3DPrGO/GCE, and PoPD-IIP/3DprGO/GCE were characterized using SEM. PS microspheres were selected as template particles due to their distinct advantages, including high water dispersion, smooth surface, and consistent size [26]. Furthermore, according to the literature [24], within the temperature range of 330 to 380 °C, PS can ultimately convert to carbon dioxide and water with ample oxygen. This process offers a convenient method for removing the template particles. As shown in Figure 1a, PS templates (1 μm in diameter) prepared using the vertical deposition method exhibited a uniform single layer on the electrode surface. Moreover, because of the abundant surface oxygen functional groups, graphene oxide (GO) nanosheets were used as the precursor for the 3DPrGO [27]. Due to the presence of the –OH and –COOH groups, GO could be stably dispersed in water and had a negative surface charge. The opposing surface charge of the GO nanosheets was crucial for the electrodeposition process. Figure 1b illustrates the rGO prepared on the electrode surface through electrodeposition, displaying typical graphene wrinkles and thin film structures. Figure 1c,d reveal that the 3DPrGO possessed a 3D porous structure, constructed with plentiful porous graphene nanosheets. Compared to the flat structure shown in Figure 1b, this 3D porous structure effectively prevented the re-stacking of graphene nanosheets. It offered a larger specific surface area and a number of exposed edge planes of graphene [28]. These factors were conducive to transferring electrons and electrolyte ions during the electrochemical reaction. Meanwhile, the 3D porous structure offered more ion-binding sites for ion-imprinted polymers, resulting in a higher rate of recognition for target ions [29]. Figure 1e,f display the SEM images of the ion-imprinted 3DPrGO/GCE before and after elution, respectively. As shown in Figure 1e, the surface of the IIP film before elution was relatively dense and smooth. In contrast, the IIP film had a rougher surface after elution, as shown in Figure 1f. The main reason was that, in removing template ions by the electrochemical peroxidation method, the IIP was also eluted off, which increased the surface roughness.

### 3.2. Electrochemical Characterization of 3DprGO/GCE Electrodes

**Figure 2:**
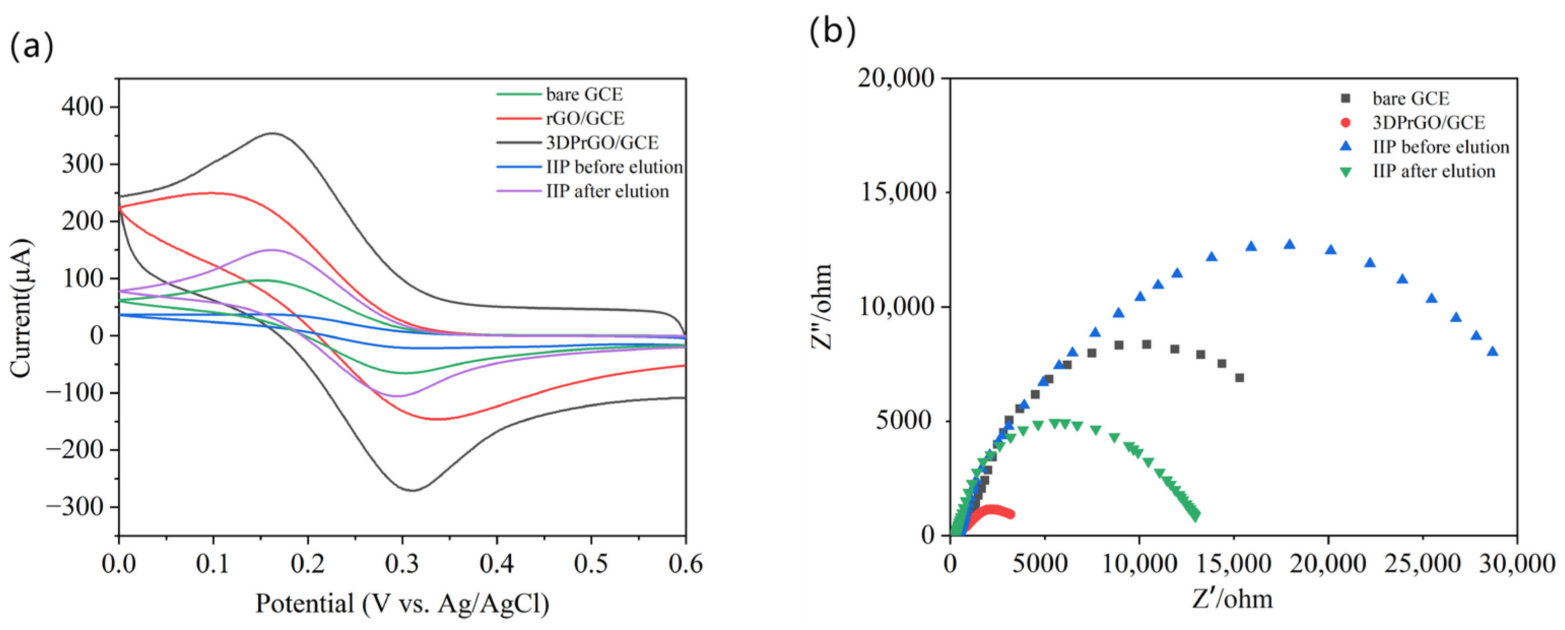
(**a**) CV scans of 3DPrGO/GCE, rGO/GCE, bare GCE, and PoPD-IIP/3DPrGO/GCE before and after elution in 0.1 mol/L KCl containing 5 mmol/L (K_3_(Fe(CN)_6_); (**b**) recorded EIS for 3DPrGO/GCE, bare GCE, and PoPD-IIP/3DPrGO/GCE before and after elution in 0.1 mol/L KCl containing 5 mmol/L (K_3_(Fe(CN)_6_).

The 3DprGO/GCE, rGO/GCE, bare GCE, and PoPD-IIP/3DPrGO/GCE before and after elution were characterized by cyclic voltammetry (CV) and electrochemical impedance spectroscopy (EIS) using (Fe(CN)_6_)^3−/4−^ as an electroactive probe. The electrochemical behaviors of each electrode were investigated in 0.1 mol/L KCl (pH 7.0) containing 5 mmol/L K_3_(Fe(CN)_6_). In the CV experiment, the scan rate was set at 100 mV/s within the potential range of 0.0 to 0.6 V. As shown in Figure 2a, the redox peak current of the bare GCE was 100 μA, whereas the peak current of the rGO/GCE (250 μA) was much higher than those at the GCE. The excellent performance of the rGO/GCE is attributed to its unique electronic structure, which provides a large number of active sites for electrochemical reactions and improves the electron transfer rate on the electrode surface [30]. The 3DPrGO/GCE exhibited a higher peak current (350 μA) than that of rGO/GCE. This is attributed to its increased surface area and open pore structures, which offer a larger electrochemical active surface area (ECSA). Consequently, this facilitates faster ion transport and a higher electrochemical reaction rate [31]. After IIP electropolymerization, however, the redox peak current significantly decreased to 37.5 μA. This may be attributed by the reduced conductivity due to the Cd(II) templates being imprinted in PoPD polymer, which inhibited electron transfer at the electrode surface. After elution of the Cd(II) template on IIP, the peak current increased to 150 μA. This demonstrates that the Cd(II) templates were removed from the IIP film and formed imprinted cavities. Through these cavities, the (Fe(CN)_6_)^3−/4−^ could directly interact with the 3DPrGO, leading to a higher peak current response than that of the bare electrode. In addition, the electrochemical active surface area (ECSA) of different electrodes were respectively calculated according to the CV redox peak values by using the Randles–Sevcik equation:

Ip = 2.69 × 10^5^n^3/2^D^1/2^υ^1/2^AC (1)

where Ip is the peak current, n is the number of transfer electrons (calculated as 1 for (Fe(CN)_6_)^3−/4−^), D is the diffusion coefficient (calculated as 6.3 × 10^−6^ cm^2^/s for (Fe(CN)_6_)^3−/4−^ in 0.10 mol/L KCl, 25 °C), v is the scan rate (v/s), A is the active surface area (cm^2^), and C is the concentration of the probe (mol/cm^3^). As a result of the calculations, the ECSA was determined to be 0.094 cm^2^ for the bare GCE, 0.235 cm^2^ for the rGO/GCE, 0.333 cm^2^ for the 3DPrGO/GCE, 0.035 cm^2^ before elution in IIP/3DPrGO/GCE, and 0.141 cm^2^ after elution in IIP/3DPrGO/GCE. The results suggest that the roughness was different between the various modified electrodes and that the 3DPrGO/GCE had the highest roughness, which may be due to the rich and open pore active edge structures of 3DPrGO.

Furthermore, EIS measurements were applied under a frequency range of 0.1 Hz~100 kHz with an amplitude of 5 mV. In Nyquist plots (Figure 2b), the semicircle diameter at a high frequency corresponds to the electron transfer resistance (Rct). The smaller the diameter, the smaller the resistance. As shown in Figure 2b, the diameter of the 3DPrGO/GCE plots was significantly smaller than that of the bare electrode, indicating a smaller Rct for the 3DPrGO/GCE than for the bare GCE. After IIP electropolymerizing without elution, the Rct increased significantly. However, after elution, the Rct decreased and became even smaller than that of the bare electrode. The EIS results are consistent with the conclusions we obtained above in the CV experiments.

### 3.3. Optimization of PoPD-IIP/3DPrGO/GCE Fabrication

#### 3.3.1. 3DPrGO Deposition Time

**Figure 3:**
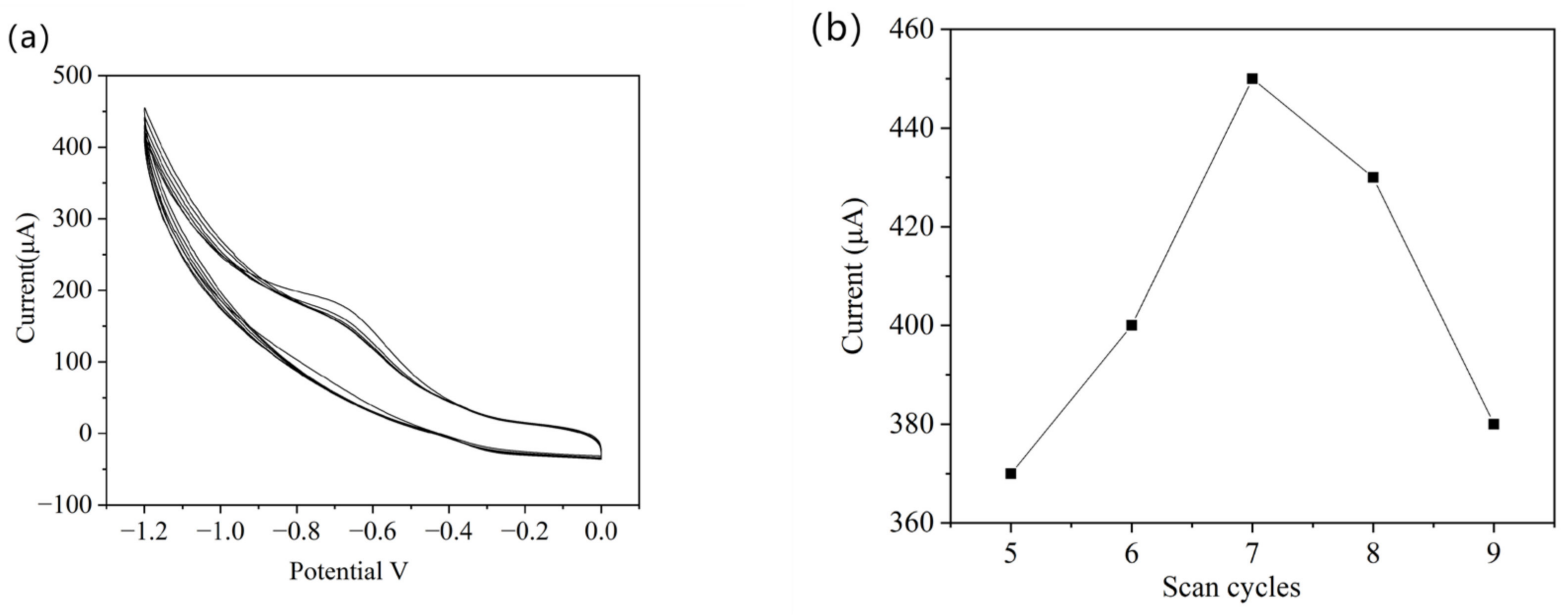
(**a**) CV curves of electrodeposited rGO on PS/GCE in 0.1 mol/L KCl solution; (**b**) effect of scan cycles on the sensitivity of 3DPrGO/GCE.

The electrodeposition time of CV scan cycles is one of the most important factors for the preparation of 3DPrGO/GCE, which directly affect the structure, density, and conductivity of 3DPrGO and the sensitivity of the sensor. Therefore, the electrochemical properties of 3DPrGO under different deposition cycles were investigated in 0.1 M KCl containing 5 mM K_3_(Fe(CN))_6_ using CV (Figure 3a). As shown in Figure 3b, the current responses increased rapidly with the increase in the scan cycle from one to seven cycles, indicating that graphene was gradually filling the void volume in the PS templates. The reason may be that the graphene nanosheets gradually formed 3D porous structures through π-π interaction with the increase in scanning cycles, providing more ECSA and reducing the internal resistance, thereby improving the electrode sensitivity [32]. However, the 3D porous graphene stacks were excessively thick and agglomerated when the scanning cycles went beyond seven cycles (Figure 1d), significantly decreasing the peak current (Figure 3b). Therefore, the optimal deposition cycle was determined to be seven cycles for 3DPrGO/GCE preparation.

#### 3.3.2. IIP Electropolymerization Time

**Figure 4:**
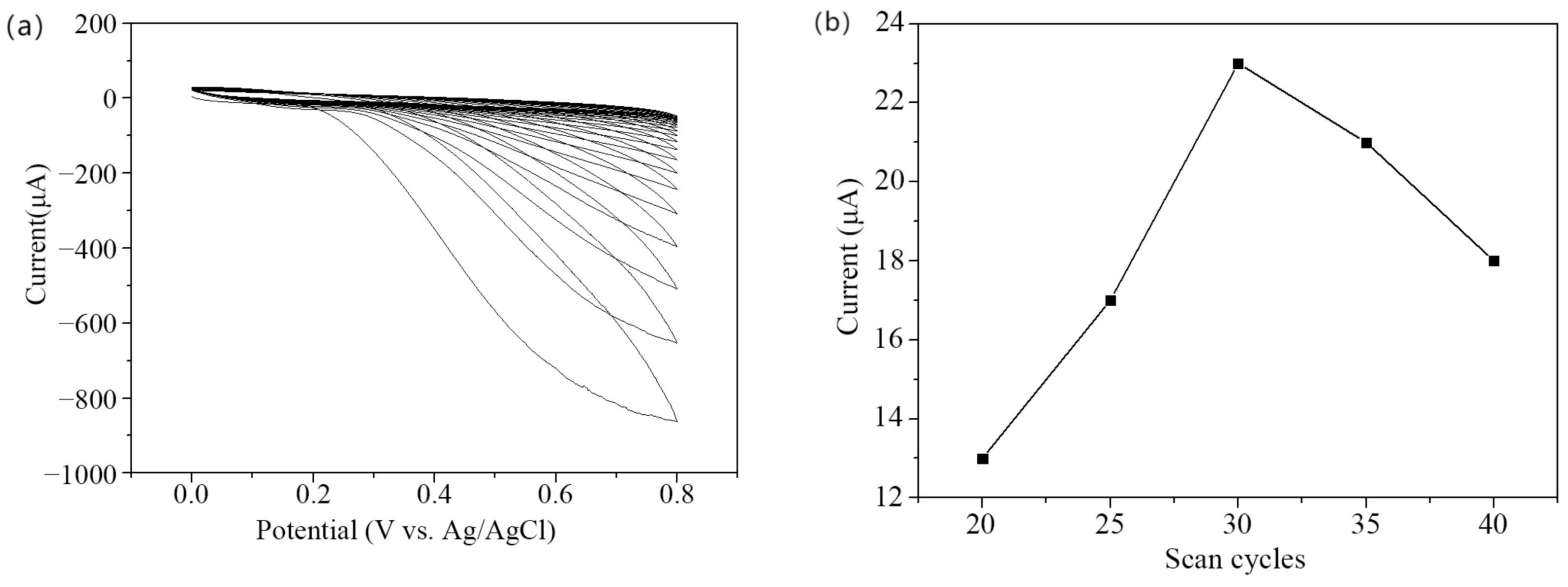
(**a**) CV curves of IIP electropolymerization on 3DPrGO/GCE in 0.1 mol/L acetate buffer solution; (**b**) effect of scan cycles on current peak values recorded from SWASV response of 30 μg/L Cd(II) (all of the obtained data are the average value of 5 measurements).

The number of CV scanning cycles in the electropolymerization time frame controls the thickness of the IIP film, which is a significant factor affecting the sensor’s performance [33]. As shown in Figure 4a, the CV curve gradually decreased and tended to be stable with increasing cycles, indicating that the oPD monomer synthesized a dense, non-conductive polymer on the electrode surface. When the CV scanning cycles were insufficient, the IIP film was too thin to create imprinted sites capable of recognizing ions [34]. Conversely, an excessive scanning cycle would have caused the IIP film to become very thick, would have caused difficulties in removing the template ions and affected the sensor’s sensitivity [35]. Therefore, the prepared PoPD-IIP/3DPrGO/GCE with different electropolymerization time frames was investigated for recording SWASV responses in 30 μg/L Cd(II) solution (pH 4.8). Figure 4b shows that the PoPD-IIP/3DPrGO/GCE had the highest Ip (peak current) when the number of scans was set to 30 cycles.

#### 3.3.3. Elution Time of Cd(II)

Complete removal is crucial for achieving the highest sensitivity and selectivity of the sensor. To ensure that the template ions in the IIP were completely removed, electrochemical oxidation was employed and the elution time was optimized. The potentiostatic method was used at 1.5 V potential to remove Cd(II), with 0.1 mol/L HCl as the eluent solution. HCl was able to break the electrostatic interactions and coordination bonds between the Cd(II) template ions and IIP. However, not all Cd(II) templates may be effectively eliminated using HCl alone, especially when the binding forces are strong. In such cases, applying a constant potential can help break the binding between Cd(II) templates and IIP, facilitating the release of the remaining templates.

**Figure 5:**
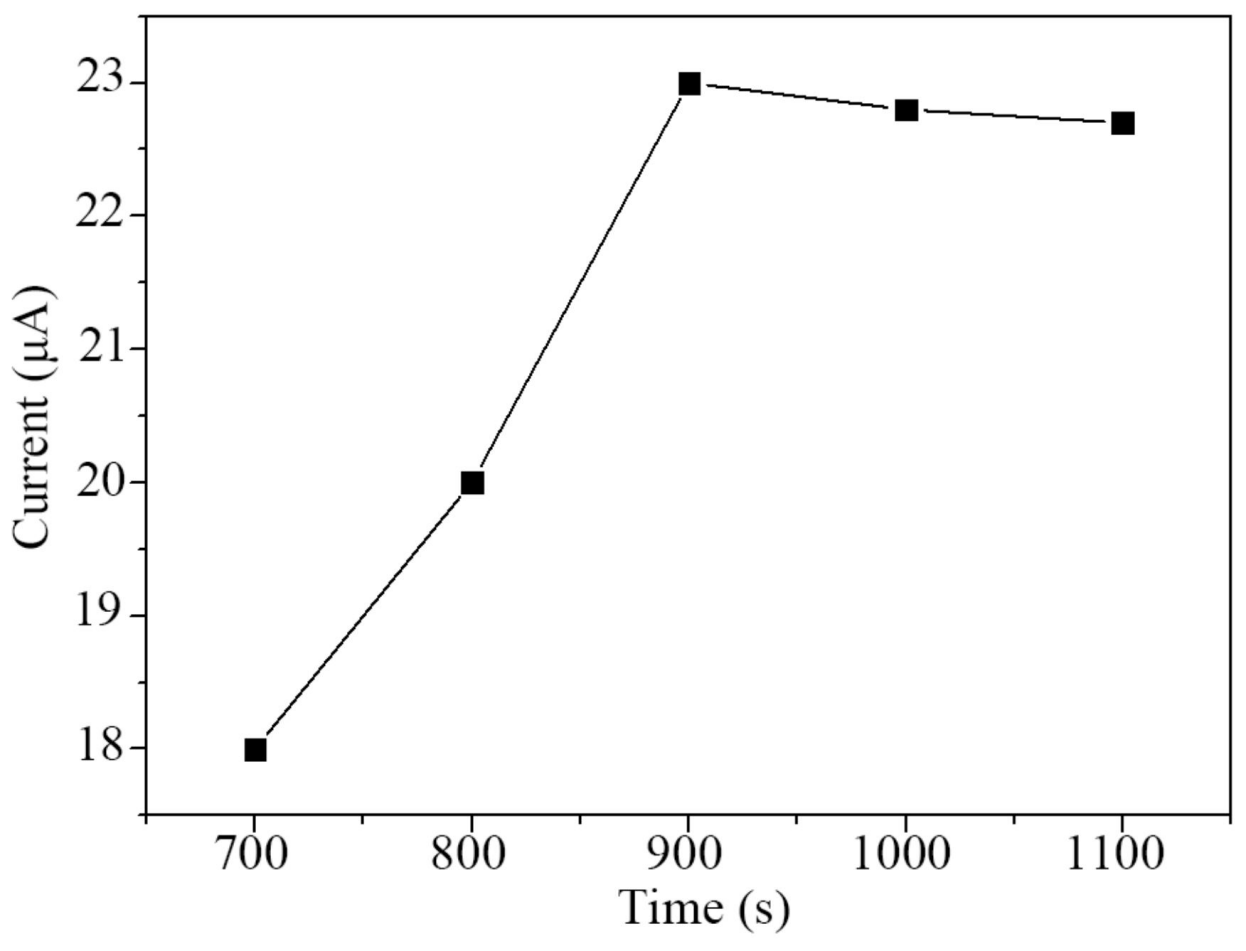
Effect of elution times on SWASV response of 30 μg/L Cd(II) (all of the obtained data are the average value of 5 measurements).

Under the optimal conditions, as mentioned before, five PoPD-IIP/3DPrGO/GCE sensors were prepared, and the elution times were set to 700 s, 800 s, 900 s, 1000 s, and 1100 s, respectively. After elution, the modified electrode was rinsed three times using deionized water to remove the residual solution and then dried with nitrogen. The SWASV responses of each sensor were investigated in 30 μg/L Cd(II) solution (pH 4.8), and the Ip values were recorded as shown in Figure 5. The Ip reached a maximum value of 23 μA at an elution time of 900 s. As the elution time increased from 900 s to 1100 s, the Ip remained stable, indicating that template ions were removed to the greatest extent possible.

### 3.4. Optimization of Conditions for Cd(II) Determination

#### 3.4.1. Effect of pH

**Figure 6:**
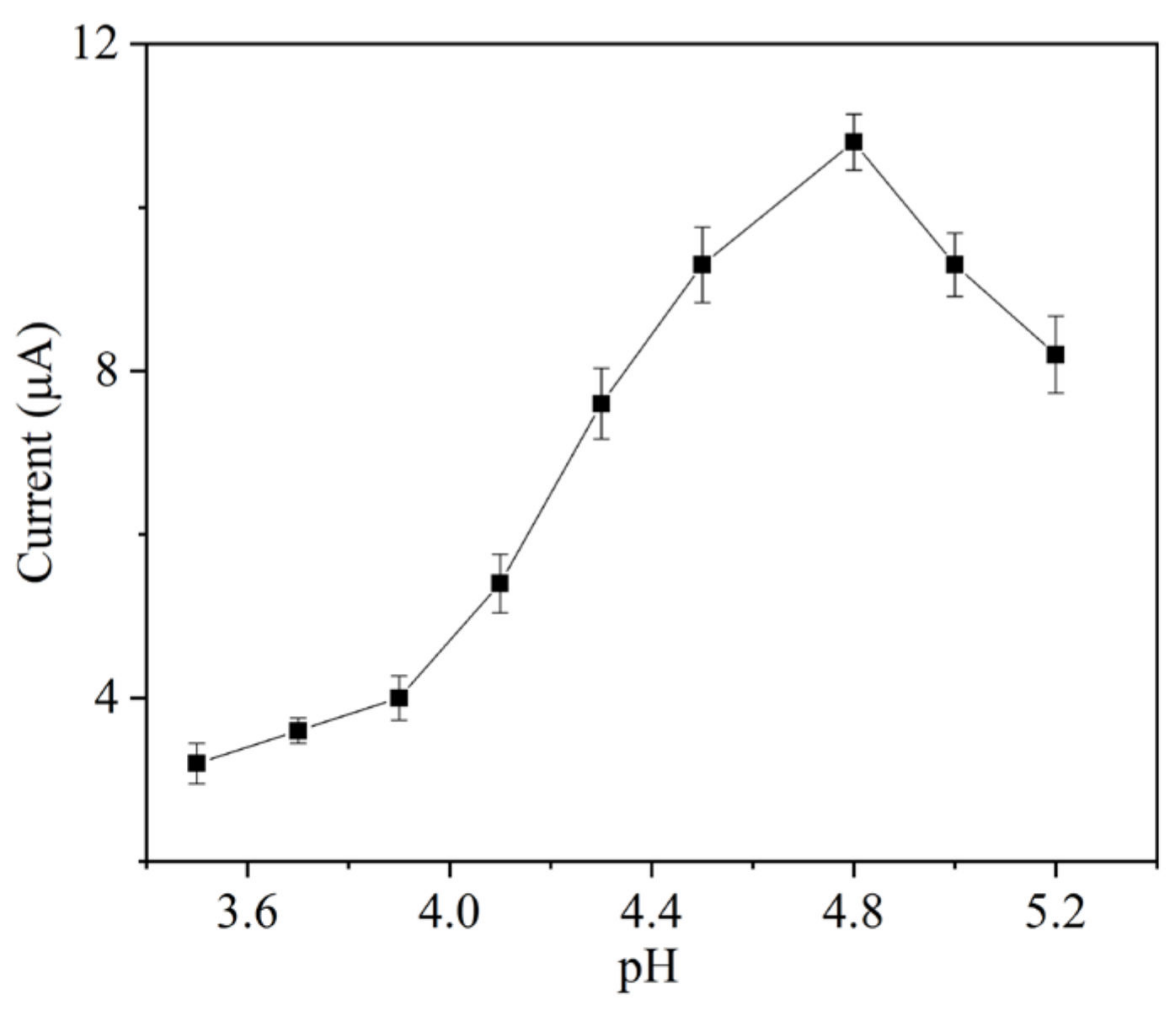
Effect of pH values on SWASV response of 10 μg/L Cd(II).

For heavy metal ions detection in water, the pH of the electrolyte affects the target ions rebinding with an ion-imprinted polymer [36]. The electrolyte solution was prepared in 0.1 mol/L acetate buffer solution (pH 4.8) containing 10 μg/L Cd(II). The optimal pH for Ip was investigated at pH values ranging from 3.5 to 5.2. As shown in Figure 6, the Ip increased as pH increased from 3.5 to 4.8, reaching the maximum at pH 4.8, and then decreased at a higher pH from 4.8 to 5.2. This result indicates that, when the pH value was 4.8, the negative charge on the surface of IIP increased the adsorption capacity of Cd(II) ions [37]. Therefore, the optimum pH value was 4.8 in this experiment.

#### 3.4.2. Effect of Accumulation Potential and Time

**Figure 7:**
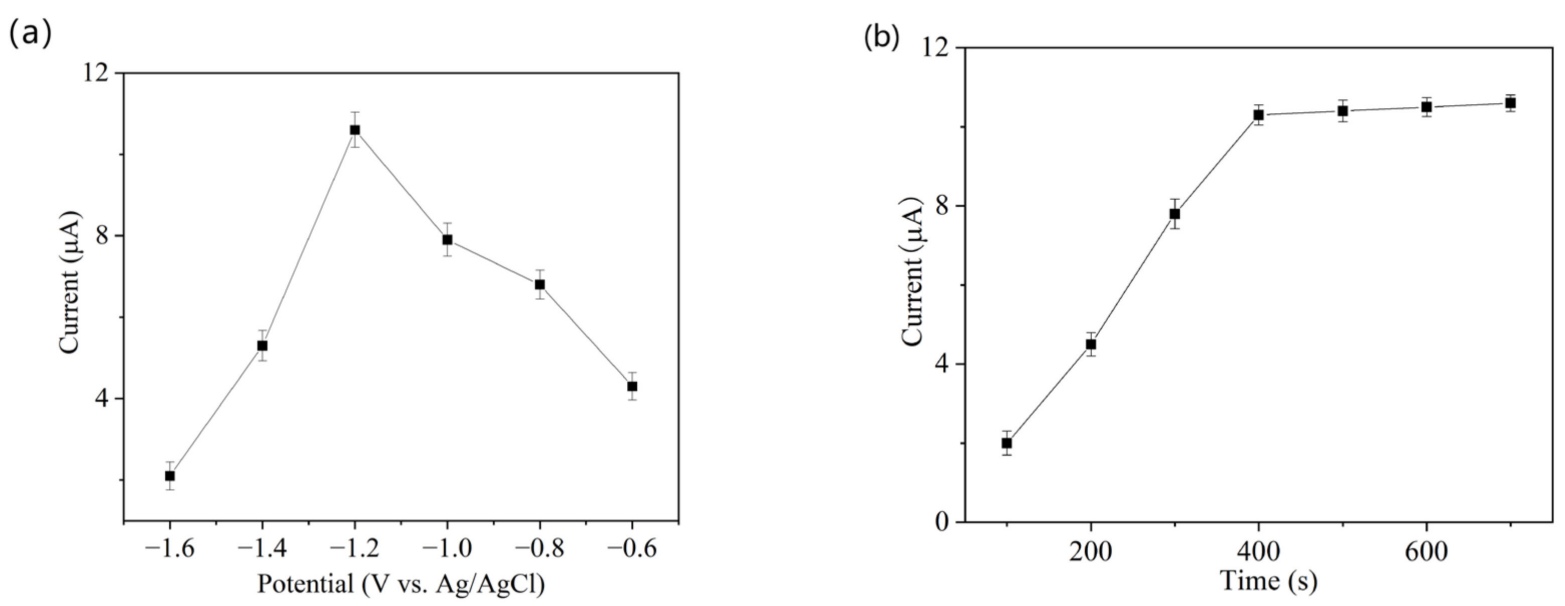
(**a**) Effect of accumulation potential on SWASV response of 10 μg/L Cd(II); (**b**) effect of accumulation times on SWASV response of 10 μg/L Cd(II).

The effect of accumulation potential on the peak current of Cd(II) was studied over a range of −0.6 to −1.6 V. As shown in Figure 7a, the Ip increased as the accumulation potential shifted from −0.8 to −1.2 V, with the highest Ip obtained at −1.2 V. The reason is that as the potential becomes more negative, more Cd(II) is reduced to Cd and deposited on the electrode surface [38]. However, the current decreased as the potential continued to decline after −1.2 V due to the more negative potential, resulting in the generation of hydrogen bubbles, which interferes with the reduction and accumulation of Cd(II) on the electrode surface [39]. Therefore, an optimal accumulation potential of −1.2 V was used in this study.

The effects of Cd(II) accumulation time on the PoPD-IIP/3DPrGO/GCE response were investigated in 10 μg/L Cd(II) solution with a pH of 4.8 at −1.2 V potential. Figure 7b shows that the Ip increased from 2 to 10.5 μA as the time increased from 100 to 400 s, and Ip plateaued as the accumulation time went beyond 400 s. The results show that, in the 10 μg/L Cd(II) solution, the binding sites on IIP reached equilibrium at 400 s. Therefore, 400 s was chosen as the optimum accumulation time.

### 3.5. Electrochemical Detection of Cd(II)

**Figure 8:**
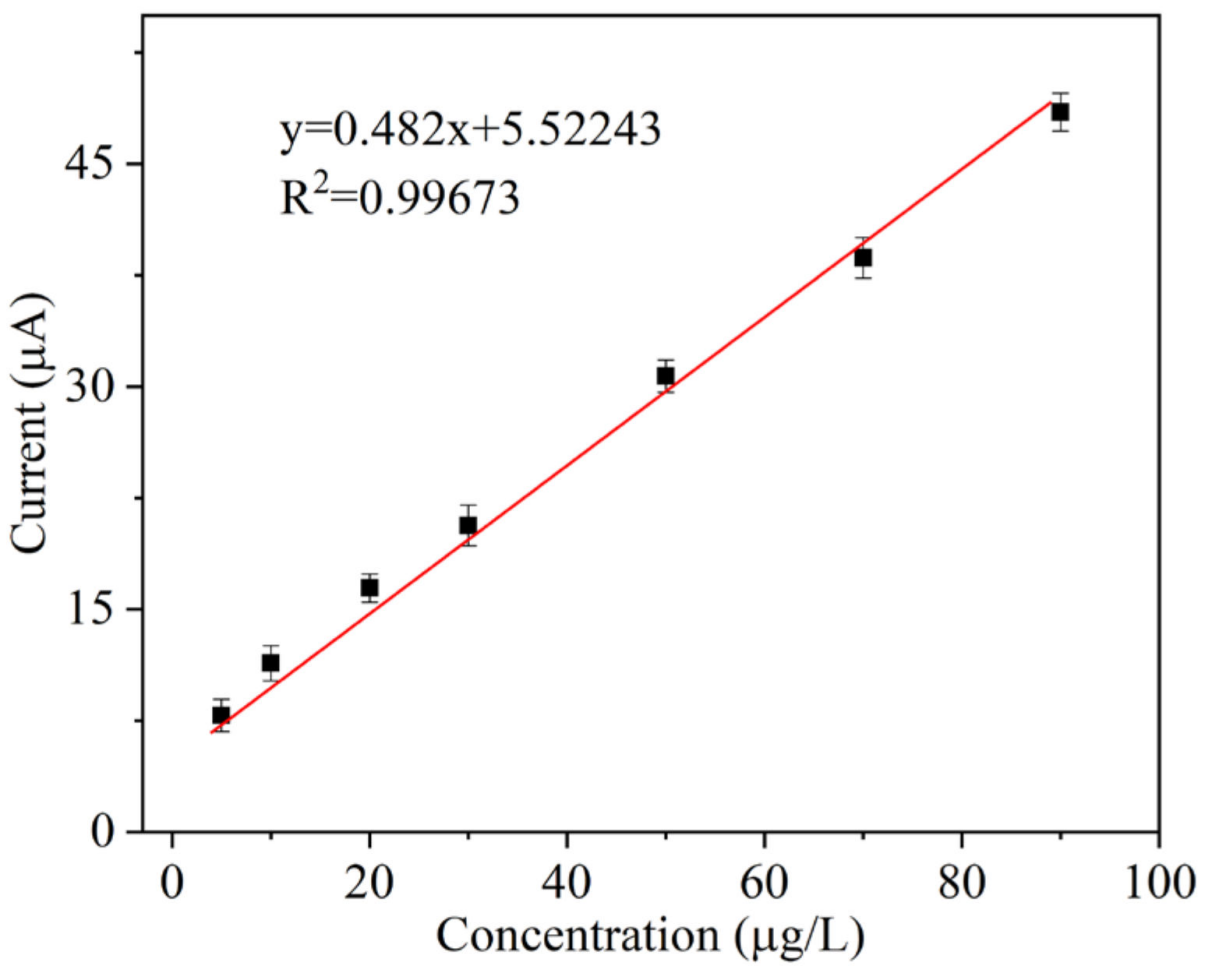
Calibration curve of PoPD-IIP/3DPrGO/GCE for Cd(II) detection.

Under optimal conditions, the SWASV method was used to determine the current responses at the PoPD-IIP/3DPrGO/GCE under different Cd(II) concentrations. As shown in Figure 8, the relation between the response current of the PoPD-IIP/3DPrGO/GCE and the Cd(II) concentration was linear, in the range of 1 to 100 μg/L. The linear equation was Ip(μA) = 0.482 C (μg/L) + 5.52243, with a correlation coefficient R2 of 0.996. The LOD was 0.11 μg/L (following the equation LOD = 3.3 σ/S, where σ is the standard deviation of the blank sample response current and S is the slope of the calibration curve). In addition, Table 1 indicates the performance of the PoPD-IIP/3DPrGO/GCE compared to that reported for the Cd(II) detection sensors. The PoPD-IIP/3DPrGO/GCE presented a superior linear range and a lower detection limit than the reported methods. This improvement can be attributed to the high conductivity of the 3DPrGO and the selectivity of the ion-imprinted polymer.

### 3.6. Selectivity, Reproducibility, and Stability of the Sensor

Other common interferent heavy metal ions in surface water, such as Cu(II), Hg(II), Zn(II), Mn(II), and Ni(II) at a concentration of 10 μg/L, were introduced to the 10 μg/L Cd(II) solution in order to assess the selectivity of the PoPD-IIP/3DPrGO/GCE. As shown in Table 2, the relative standard deviations (RSDs) for each detection were less than 5%, indicating that these heavy metal ions had no apparent interference in Cd(II) detection.

**Figure 9:**
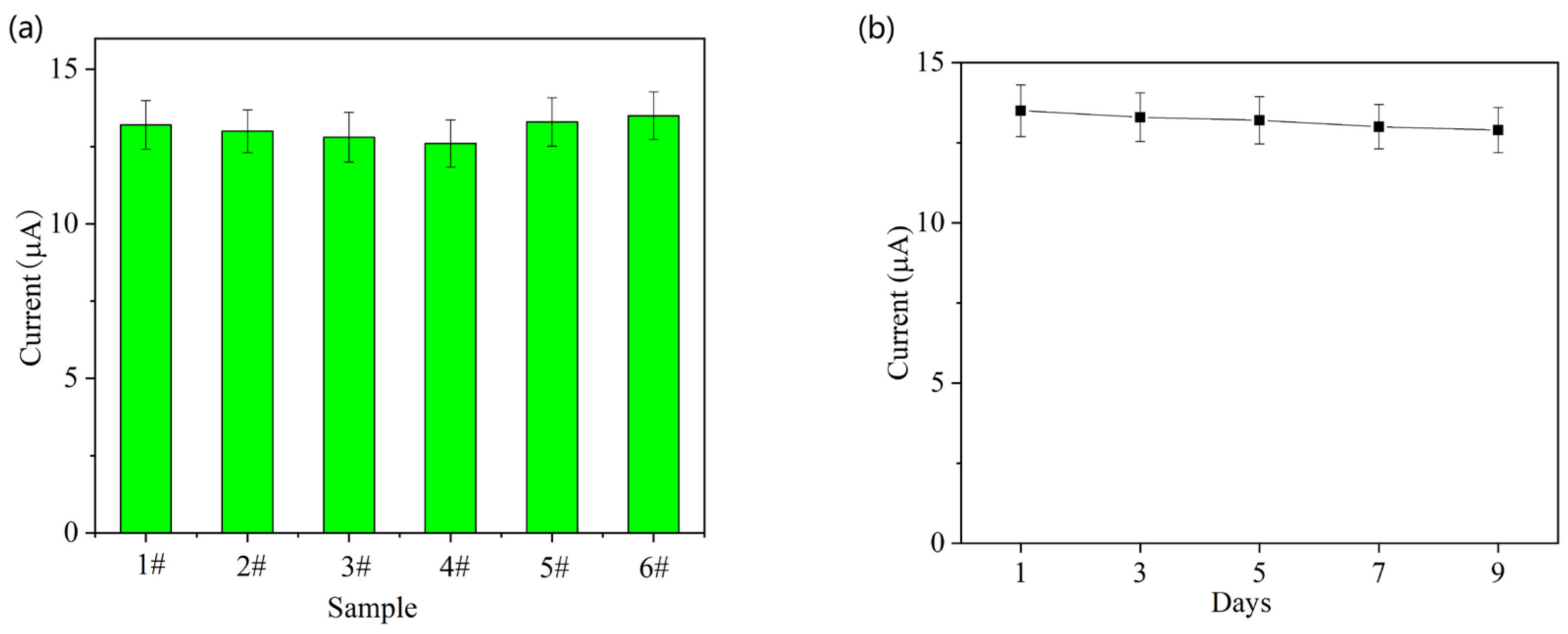
(**a**) SWASV responses of 10 μg/L Cd(II) obtained with six sensors. (**b**) SWASV response within 9 days for the same sensor.

Six PoPD-IIP/3DPrGO/GCE sensors were fabricated under the same conditions to investigate the reproducibility. The SWASV responses of the six sensors to 10 μg/L Cd(II) are compared in Figure 9a, which shows that an RSD of 2.8% (*n* = 6) was obtained, indicating good reproducibility of the material modification on GCE.

The stability of the PoPD-IIP/3DPrGO/GCE was investigated by measuring its SWASV response to Cd(II) after storing at room temperature for 9 days. As shown in Figure 9b, the current responses retained 98.5% of its initial response, which indicates that the prepared sensor had good stability.

### 3.7. Detection of Cd(II) in Real Samples

To validate the practicality of the PoPD-IIP/3DPrGO/GCE sensor detecting Cd(II) in actual samples, the water samples collected from three rivers in Beijing were used for testing. The standard addition method was performed for all of the samples, and the same pre-treatment was applied to each sample to obtain the most accurate results. The results are summarized in Table 3. The recoveries were 97%, 98.6%, and 99.6%, respectively. Based on these results, it can be concluded that this sensor is reliable and effective for the determination of Cd(II) in real samples.

## 4. Conclusions

Three-dimensional porous graphene oxide (3DPrGO) nanostructures were synthesized in situ by electrochemical reduction coupled with the PS colloidal crystal template assistant method, where no toxic reagents and only a relatively low temperature of 350 °C were used for template removal. The as-prepared 3DPrGO materials were functionalized with IIP to develop a new electrochemical sensor for trace Cd(II) detection in water. The present IIP/3DPrGO/GCE sensor showed rich porous structures, increased electroactive surface area, many more recognition sites, and enhanced sensitivity towards Cd(II) detection. Furthermore, the sensor exhibited excellent selectivity, repeatability, and stability and displayed a wide linear detection range, low detection limit, and satisfying recovery results in real water sample measurement. The sensor fabrication was facile, safe, and effective and would contribute to a powerful platform for rapid and sensitive analysis of Cd(II) in water.

## Figures and Tables

**Table 1:** Comparison of the performance of PoPD-IIP/3DPrGO/GCE with other reported Cd(II) sensors.

Modified Electrode	Linear Range (μg/L)	Detection Limit (μg/L)	References
Au/rGOCNT/Bi	20–200	0.6	[40]
CUiO-66/Bi/GCE	10–50	1.16	[41]
ZJU-77/Nafion/GCE	0–60	0.12	[42]
PoPD-IIP/rGO/GCE	1–50	0.13	[23]
PoPD-IIP/3DPrGO/GCE	1–100	0.11	This work

**Table 2:** Cross-sensitivity of PoPD-IIP/3DPrGO/GCE against other heavy metal ions.

Interference Ion	Concentration (μg/L)	RSD (%)
Cu^2+^	10	3.91%
Hg^2+^	10	4.88%
Mn^2+^	10	1.61%
Zn^2+^	10	2.82%
Ni^2+^	10	2.28%

**Table 3:** Test results of cadmium ions in actual water samples.

Water Sample	Cd(II) Added (μg/L)	Cd(II) Found (μg/L)	Recovery (%)	RSD (%, *n* = 3)
Sample 1	1.00	0.97	97	4.3
Sample 2	10.00	9.86	98.6	3.5
Sample 3	100.00	99.6	99.6	5.7

## Data Availability

Data are contained within the article.

## References

[1] Irfan M., Liu X., Hussain K., Mushtaq S., Cabrera J., Zhang P. (2023). The global research trend on cadmium in freshwater: A bibliometric review. Environ. Sci. Pollut. Res..

[2] Wang R., Sang P., Guo Y., Jin P., Cheng Y., Yu H., Xie Y., Yao W., Qian H. (2023). Cadmium in food: Source, distribution and removal. Food Chem..

[3] Naksen P., Boonruang S., Yuenyong N., Lee H.L., Ramachandran P., Anutrasakda W., Amatatongchai M., Pencharee S., Jarujamrus P. (2022). Sensitive detection of trace level Cd (II) triggered by chelation enhanced fluorescence (CHEF) “turn on”: Nitrogen-doped graphene quantum dots (N-GQDs) as fluorometric paper-based sensor. Talanta.

[4] Karlidag N.E., Toprak M., Demirel R., Zaman B.T., Bakirdere S. (2022). Development of copper nanoflowers based dispersive solid-phase extraction method for cadmium determination in shalgam juice samples using slotted quartz tube atomic absorption spectrometry. Food Chem..

[5] Chen B., Zhang L., He M., Hu B. (2020). Cd (II) imprinted polymer modified silica monolithic capillary microextraction combined with inductively coupled plasma mass spectrometry for the determination of trace Cd (II) in biological samples. Spectrochim. Acta Part B At. Spectrosc..

[6] Yuan Y., Wu Y., Wang H., Tong Y., Sheng X., Sun Y., Zhou X., Zhou Q. (2020). Simultaneous enrichment and determination of cadmium and mercury ions using magnetic PAMAM dendrimers as the adsorbents for magnetic solid phase extraction coupled with high performance liquid chromatography. J. Hazard. Mater..

[7] Shafqat S.S., Rizwan M., Batool M., Shafqat S.R., Mustafa G., Rasheed T., Zafar M.N. (2023). Metal organic frameworks as promising sensing tools for electrochemical detection of persistent heavy metal ions from water matrices: A concise review. Chemosphere.

[8] Ren Y., Xu Y. (2023). Three-dimensional graphene/metal-organic framework composites for electrochemical energy storage and conversion. Chem. Commun..

[9] Lang W., Yue C., Dang M., Wang G., Chen Y., Hu F., Liu Z., Shu J. (2023). Germanium decorated on three dimensional graphene networks as binder-free anode for Li-ion batteries. J. Power Sources.

[10] Ion-Ebrasu D., Andrei R.D., Enache S., Caprarescu S., Negrila C.C., Jianu C., Enache A., Boerasu I., Carcadea E., Varlam M. (2021). Nitrogen Functionalization of CVD Grown Three-Dimensional Graphene Foam for Hydrogen Evolution Reactions in Alkaline Media. Materials.

[11] Cheng Y., Wang K., Qi Y., Liu Z. (2022). Chemical Vapor Deposition Method for Graphene Fiber Materials. Acta Phys.-Chim. Sin..

[12] Li W., Wu N., Che S., Sun L., Liu H., Ma G., Wang Y., Xu C., Li Y. (2023). Polyurethane foam-supported three-dimensional interconnected graphene nanosheets network encapsulated in polydimethylsiloxane to achieve significant thermal conductivity enhancement. Front. Mater. Sci..

[13] Jin L., Wang P., Cao W., Song N., Ding P. (2022). Isolated Solid Wall-Assisted Thermal Conductive Performance of Three-Dimensional Anisotropic MXene/Graphene Polymeric Composites. ACS Appl. Mater. Interfaces.

[14] Salazar-Aguilar A.D., Ivan Rodriguez-Rodriguez J., Pineiro-Garcia A., Tristan F., Judith Labrada-Delgado G., Meneses-Rodriguez D., Magdalena Vega-Diaz S. (2021). Layer-by-Layer Method to Prepare Three-Dimensional Reduced Graphene Materials with Controlled Architectures Using SiO_2_ as a Sacrificial Template. Ind. Eng. Chem. Res..

[15] Chen C.-M., Chen C.-M. (2016). Template-Directed Macroporous ‘Bubble’ Graphene Film for the Application in Supercapacitors. Surface Chemistry and Macroscopic Assembly of Graphene for Application in Energy Storage.

[16] Chen Z., Zhang Y., Zhang J., Zhou J. (2019). Electrochemical Sensing Platform Based on Three-Dimensional Holey Graphene for Highly Selective and Ultra-Sensitive Detection of Ascorbic Acid, Uric Acid, and Nitrite. J. Electrochem. Soc..

[17] Feng L., Qin W., Wang Y., Gu C., Li X., Chen J., Chen J., Qiao H., Yang M., Tian Z. (2023). Ti3C2Tx MXene/Graphene/AuNPs 3D porous composites for high sensitivity and fast response glucose biosensing. Microchem. J..

[18] Gao H., Ma Y., Song P., Yang Z., Wang Q. (2021). Three-dimensional reduced graphene oxide/cobaltosic oxide as a high-response sensor for triethylamine gas at room temperature. Mater. Sci. Semicond. Process..

[19] Velusamy K., Periyasamy S., Kumar P.S., Rangasamy G., Pauline J.M.N., Ramaraju P., Mohanasundaram S., Dai-Viet Nguyen V. (2022). Biosensor for heavy metals detection in wastewater: A review. Food Chem. Toxicol..

[20] Metwally M.G., Benhawy A.H., Khalifa R.M., El Nashar R.M., Trojanowicz M. (2021). Application of Molecularly Imprinted Polymers in the Analysis of Waters and Wastewaters. Molecules.

[21] Yola B.B., Bekerecioglu S., Polat I., Atar N., Yola M.L. (2023). A novel electrochemical detection method for butylated hydroxyanisole (BHA) as an antioxidant: A BHA imprinted polymer based on a nickel ferrite@graphene nanocomposite and its application. Analyst.

[22] Isotalo T.J., Tian Y.-L., Konttinen M.P., Maasilta I.J. (2014). Statistical characterization of self-assembled colloidal crystals by single-step vertical deposition. Colloids Surf. A Physicochem. Eng. Asp..

[23] Wang J., Hu J., Hu S., Gao G., Song Y. (2020). A Novel Electrochemical Sensor Based on Electropolymerized Ion Imprinted PoPD/ERGO Composite for Trace Cd(II) Determination in Water. Sensors.

[24] Lu Y.-C., Tseng P.-C., Yang M.-J., Wang C.-J., Ling Y.-C., Lin C.-F., Hsueh H.-Y. (2022). Fabrication of Gyroid-Structured Metal/Semiconductor Nanoscaffolds with Ultrasensitive SERS Detection via Block Copolymer Templating. Adv. Opt. Mater..

[25] Ding Q., Li C., Wang H., Xu C., Kuang H. (2021). Electrochemical detection of heavy metal ions in water. Chem. Commun..

[26] Wang Y., Huang Y., Wang B., Fang T., Chen J., Liang C. (2016). Three-dimensional porous graphene for simultaneous detection of dopamine and uric acid in the presence of ascorbic acid. J. Electroanal. Chem..

[27] Huang C.-Y., Lin Y.-C., Chung J.H.Y., Chiu H.-Y., Yeh N.-L., Chang S.-J., Chan C.-H., Shih C.-C., Chen G.-Y. (2023). Enhancing Cementitious Composites with Functionalized Graphene Oxide-Based Materials: Surface Chemistry and Mechanisms. Int. J. Mol. Sci..

[28] Huang Y., Li P., Han Y., Zhang Y., Han L. (2022). Controllable surface engineered three-dimensional porous graphene based electrode for ultrasensitive flexible electrochemical sensing. Appl. Surf. Sci..

[29] Yang L., Xu B., Ye H., Zhao F., Zeng B. (2017). A novel quercetin electrochemical sensor based on molecularly imprinted poly(para-aminobenzoic acid) on 3D Pd nanoparticles-porous graphene-carbon nanotubes composite. Sens. Actuators B Chem..

[30] Renjini S., Abraham P., Kumary V.A., Chithra P.G., Sreevalsan K. (2022). Review-Progress on Carbon-Based Electrochemical Sensors for Epinephrine and Norepinephrine. J. Electrochem. Soc..

[31] Yu X., Zhang M., Yuan W., Shi G. (2015). A high-performance three-dimensional Ni-Fe layered double hydroxide/graphene electrode for water oxidation. J. Mater. Chem. A.

[32] Jiang L., Chen J., Wang Y., Sun K., Liu F., Lai Y. (2018). Graphene-Sb2Se3 thin films photoelectrode synthesized by in situ electrodeposition. Mater. Lett..

[33] Liu H., Xie M., Pan B., Li N., Zhang J., Lu M., Luo J., Wang H. (2022). In-situ intercalated pyrolytic graphene/serpentine hybrid as an efficient lubricant additive in paraffin oil. Colloids Surf. A Physicochem. Eng. Asp..

[34] Kor K., Zarei K. (2016). Development and characterization of an electrochemical sensor for furosemide detection based on electropolymerized molecularly imprinted polymer. Talanta.

[35] Crapnell R.D., Hudson A., Foster C.W., Eersels K., van Grinsven B., Cleij T.J., Banks C.E., Peeters M. (2019). Recent Advances in Electrosynthesized Molecularly Imprinted Polymer Sensing Platforms for Bioanalyte Detection. Sensors.

[36] Zhang Q., Wu J., Luo X. (2016). Facile preparation of a novel Hg(II)-ion-imprinted polymer based on magnetic hybrids for rapid and highly selective removal of Hg(II) from aqueous solutions. Rsc Adv..

[37] Zhu F., Li L., Xing J. (2017). Selective adsorption behavior of Cd(II) ion imprinted polymers synthesized by microwave-assisted inverse emulsion polymerization: Adsorption performance and mechanism. J. Hazard. Mater..

[38] Garkani-Nejad Z., Akbari Javar H., Mahmoudi-Moghaddam H. (2022). An efficient sensor for simultaneous determination of Hg (II) and As (III) using a carbon paste electrode modified with needle-shaped Pt-doped NiCo2O4 nanograss. Sens. Actuators B Chem..

[39] Solís J.C., Galicia M. (2020). High Performance of MWCNTs—Chitosan Modified Glassy Carbon Electrode for Voltammetric Trace Analysis of Cd(II). Int. J. Electrochem. Sci..

[40] Xuan X., Park J.Y. (2018). A miniaturized and flexible cadmium and lead ion detection sensor based on micro-patterned reduced graphene oxide/carbon nanotube/bismuth composite electrodes. Sens. Actuators B-Chem..

[41] Ding Y., Wei F., Dong C., Li J., Zhang C., Han X. (2021). UiO-66 based electrochemical sensor for simultaneous detection of Cd(II) and Pb(II). Inorg. Chem. Commun..

[42] Li Y., Xia T., Zhang J., Cui Y., Li B., Yang Y., Qian G. (2019). A manganese-based metal-organic framework electrochemical sensor for highly sensitive cadmium ions detection. J. Solid State Chem..

